# Integrated High-Throughput Targeted Metabolomics and Machine Learning for Early Prediction and Prevention of Postoperative Delirium in Older Adult Surgical Patients: Prospective Multicenter Cohort Study

**DOI:** 10.2196/78495

**Published:** 2026-04-30

**Authors:** Gengrui Zhong, Xiaoli Huang, Congye Li, Deqiang Wang, Dingding Huang, Menghan Sun, Quanhong Zhou, Yong Guo

**Affiliations:** 1 Department of Critical Care Medicine, The Sixth People's Hospital Affiliated to Shanghai Jiao Tong University School of Medicine, 600 Yishan Road, Shanghai, 200233, China, 86 15921172011; 2 Department of Critical Care Medicine, Jinshan District Central Hospital, Shanghai, China; 3 Department of Critical Care Medicine, Fengxian District Central Hospital, Shanghai, China; 4 Department of Anesthesiology, Fengxian District Central Hospital, Shanghai, China; 5 Department of Critical Care Medicine, Shanghai Eighth People's Hospital, Shanghai, China

**Keywords:** high-throughput targeted metabolomics, machine learning, prediction and prevention, postoperative delirium, older adult surgical patients

## Abstract

**Background:**

Postoperative delirium (POD) is a common and severe complication in older adult patients with hip fracture, yet its pathogenesis remains unclear.

**Objective:**

This study aimed to develop a predictive model for POD following hemiarthroplasty in older adult patients by integrating high-throughput targeted metabolomics and machine learning.

**Methods:**

In this prospective multicenter cohort study, 260 older adult patients undergoing hemiarthroplasty for hip fracture were enrolled. Preoperative serum samples were analyzed via high-throughput targeted metabolomics. Differential metabolites were screened using random forest (RF) and least absolute shrinkage and selection operator regression. Predictive models were constructed using gradient boosting, logistic regression, and RF, with performance evaluated using receiver operating characteristic curves and the area under these curves (area under the receiver operating characteristic curve [AUC]).

**Results:**

Absolute quantification of 201 metabolites revealed 41 (20.4%) significantly differentially expressed metabolites. RF and least absolute shrinkage and selection operator regression identified 16 candidate biomarkers. The logistic regression model demonstrated optimal performance, achieving an AUC of 0.855 (95% CI 0.800‐0.910) in the overall cohort. Upon 7:3 partitioning into training and test sets, the model maintained robust predictive accuracy, with AUCs of 0.844 and 0.856, respectively.

**Conclusions:**

Integration of preoperative metabolomics and machine learning enabled accurate prediction of POD in older adult patients with hip fracture, facilitating personalized risk stratification and tailored clinical management.

## Introduction

Postoperative delirium (POD) is a common and severe neuropsychiatric syndrome affecting 10% to 50% of older adult surgical patients, particularly within the first 48 hours postoperatively. Characterized by acute fluctuations in attention, cognitive impairment, and altered consciousness, POD significantly prolongs intensive care unit stays, increases hospitalization costs, and elevates mortality rates [1]. Long-term consequences include persistent cognitive decline and an elevated risk of dementia [2,3]. The pathogenesis of POD remains elusive, with hypotheses spanning neuroinflammation, neurotransmitter imbalances, metabolic dysregulation, and sleep-wake cycle disruptions [4]. Notably, preoperative cognitive impairment, advanced age, and perioperative stressors such as surgery duration and blood transfusions have been identified as independent risk factors [5,6]. Given the global aging population and rising surgical demand, there is an urgent need for early diagnostic tools and preventive strategies to mitigate POD’s clinical and economic burdens.

Current diagnostic approaches rely heavily on clinical observation, which lacks sensitivity and specificity, particularly in the early stages of POD [7]. While metabolic dysregulation has emerged as a key feature of POD, traditional biomarkers lack predictive power. Metabolomics—a systems biology approach analyzing small-molecule metabolites—offers unprecedented opportunities to elucidate underlying pathophysiological pathways [8]. For instance, our preliminary studies revealed significant disturbances in polyunsaturated fatty acids, energy metabolism, oxidative stress, and amino acid imbalances in postoperative patients [9,10]. Furthermore, systemic inflammation markers (eg, interleukin-6 and C-reactive protein) and β-amyloid peptides correlated with POD onset in vulnerable populations [11]. However, existing research has yet to integrate high-throughput targeted metabolomics with artificial intelligence to develop predictive models capable of identifying at-risk individuals preoperatively [12].

Surgery-induced systemic inflammation is a core driver of POD, wherein metabolites act as dual-effect mediators—either amplifying inflammation or providing neuroprotection. Proinflammatory lipid mediators derived from arachidonic acid, such as prostaglandin E2, can directly damage hippocampal synapses [13,14], whereas preoperative deficiencies in gut microbiota–derived short-chain fatty acids such as propionate impair the suppression of neuroinflammation via the nuclear factor kappa-light-chain-enhancer of activated B cell pathway [15]. Concurrently, imbalances in neurotransmitter systems significantly contribute to cognitive dysfunction. Inadequate availability of choline, a precursor for acetylcholine synthesis, combined with metabolic alterations in γ-aminobutyric acid precursors such as L-serine, exacerbates postoperative neuropsychiatric symptoms [13]. Furthermore, the age-related decline in cerebral metabolic reserve predisposes older adult patients to neuronal energy failure under surgical stress. Disruptions in the tricarboxylic acid (TCA) cycle lead to lactate accumulation, and deficiencies in branched-chain amino acids—key substrates for neuronal energy metabolism—compromise neurotrophic support, establishing a pathological cycle of “energy substrate exhaustion–metabolic waste accumulation” [16]. These mechanisms underscore the value of metabolites as “molecular sensors” in POD pathophysiology, providing a theoretical foundation for our targeted metabolomics approach focused on inflammation, neurotransmission, and energy metabolism.

Building on prior work characterizing metabolic disturbances in geriatric perioperative patients [17-19], this study represents a paradigm shift by integrating high-throughput targeted metabolomics with machine learning to develop a clinically actionable prediction model for POD in older adult patients undergoing hip hemiarthroplasty. Unlike conventional approaches that rely on single biomarkers or broad genomic frameworks [19,20], our targeted metabolomics platform enables high quantitative accuracy and reproducibility, thereby facilitating clinical translation [21-23]. By moving beyond traditional physiological parameters and leveraging machine learning to integrate multiple metabolite markers, we aim to overcome the limitations of single-biomarker strategies and conventional statistical models.

Therefore, the primary objective of this multicenter prospective cohort study was to identify preoperative serum metabolite signatures associated with POD by using high-throughput targeted metabolomics. Furthermore, we sought to develop and validate a machine learning–based predictive model that integrates these metabolite biomarkers to enable early risk stratification and individualized prevention of POD in older adult surgical patients. This integrative approach not only aims to advance the mechanistic understanding of POD but also holds potential for clinical implementation in perioperative care.

## Methods

### Study Design

This prospective observational multicenter cohort study was conducted from June 2021 to January 2024, with patient recruitment from June 2021 to December 2023 and follow-up completion in January 2024. In this study, 586 older adult patients (aged ≥60 years) scheduled for hemiarthroplasty due to hip fractures were initially enrolled. Inclusion criteria were age of 60 years or above and undergoing total hip replacement under uniform general anesthesia. Exclusion criteria were American Society of Anesthesiologists physical status classification of grade III or higher, Mini-Mental State Examination (MMSE) score below 23, history of central nervous system (CNS) disorders or psychiatric diseases, current use of sedatives or antidepressants, preoperative biochemical abnormalities indicating renal dysfunction or active hepatic disease, preexisting diabetes mellitus, history of neurosurgical or cardiac surgery, refusal to complete study protocols or inability to comprehend the study language, severe auditory or visual impairment, illiteracy, Parkinson disease, and alcohol or substance dependence. All baseline data (demographics and clinical characteristics), preoperative serum samples, and postoperative outcomes (POD incidence and cognitive function) were collected prospectively according to a predefined protocol without retrospective data retrieval.

### Ethical Considerations

All experimental procedures in this study were approved by the Ethics Committee of the Sixth People’s Hospital Affiliated to Shanghai Jiao Tong University School of Medicine (approval number 2021-YS-237). The study was conducted in accordance with the Declaration of Helsinki. Written informed consent was obtained from each participant or their legally authorized representative prior to enrollment. To protect participant privacy and confidentiality, all personal identifiers were removed, and data were anonymized prior to analysis. Access to study data was restricted to authorized research personnel only. No financial compensation was provided to participants. All study procedures in this research have been registered at the Chinese Clinical Trial Registry [24] with the registration number ChiCTR-CPC-15006141.

### Dietary Management

All patients in the experimental group received a light diet preoperatively. Semiliquid feeding was initiated 6 hours after general anesthesia, followed by a gradual transition to a light regular diet.

### Anesthetic Protocol

Patients underwent standardized general anesthesia for hemiarthroplasty. Premedication included intramuscular midazolam (3 mg). Anesthesia induction was performed with intravenous fentanyl (0.1 mg), propofol (1.5 mg/kg), and vecuronium (0.1 mg/kg). Maintenance anesthesia was administered via a semiclosed circuit system using sevoflurane (1‐1.5 maximum allowable concentration) and oxygen (1 L/min). Continuous intraoperative monitoring included pulse oximetry, invasive arterial blood pressure waveform, end-tidal partial pressure of carbon dioxide, and 3-lead electrocardiography.

### Surgical Procedure

Hemiarthroplasty involved replacement of the fractured femoral head with a metallic prosthesis and insertion of a metal stem into the femoral shaft to enhance implant stability.

### Biospecimen Collection

Preoperative blood samples were collected on the day of the surgery prior to entering the operating room. Postoperative samples were obtained immediately upon transfer from the postanesthesia care unit. Serum was cryopreserved for subsequent high-throughput targeted metabolomics profiling.

### POD Diagnosis

Among the 260 enrolled patients undergoing total hip replacement, POD was assessed twice daily (8 AM to 8 PM on postoperative days 1‐3) using the Confusion Assessment Method (CAM) [25,26] as recommended by the *Diagnostic and Statistical Manual of Mental Disorders, Fourth Edition*. Diagnostic criteria were (1) acute onset with fluctuating symptoms, (2) inattention, (3) disorganized thinking, and (4) altered consciousness. POD was confirmed if criteria 1 and 2 were present along with either criterion 3 or 4. Patients were categorized into POD and non-POD groups.

The CAM (POD diagnosis) was conducted by research nurses certified in the CAM scale. Before study initiation, all involved nurses completed certified training for the Chinese version of the CAM scale (certificate numbers archived). The assessment results were reviewed, and the POD diagnosis was confirmed by the designated psychiatrists (see the Perioperative Cognitive Assessment section), thereby avoiding potential bias from nurses’ subjective judgments.

### Clinical Data Collection

Baseline demographic data, anesthesia- and surgery-related parameters, and other clinical variables were systematically documented. Clinical data collection was performed by research coordinators. Each center designated 1 coordinator (with a master’s degree or higher in nursing or a related field) responsible for recording baseline characteristics (age, sex, and BMI) and surgical and anesthesia parameters (operation duration and anesthesia duration). Data entry was monitored weekly by a clinical research associate who spot-checked 20% of cases to ensure accuracy.

### Observer Selection and Training for Clinical Assessments

#### CAM Assessors (Research Nurses)

All nurses completed standardized training on the Chinese CAM version led by psychiatrists and obtained certification through theoretical and practical examinations to ensure accurate identification of core delirium symptoms. Only certified nurses participated.

#### MMSE Assessors (Psychiatrists)

Each center designated 1 psychiatrist with 5 years or more of geriatric neuropsychological experience to conduct all MMSE evaluations, minimizing interassessor variability and ensuring consistent scoring practices.

### High-Throughput Targeted Metabolomics Platform

Serum samples were analyzed using the Q300 metabolite assay kit (Metabo-Profile) [27]. Sample processing, including protein precipitation and derivatization, was automated on an Eppendorf epMotion workstation. Metabolite separation and detection were performed using ultrahigh-performance liquid chromatography (UPLC) coupled with tandem mass spectrometry (ACQUITY UPLC Xevo TQ-S system; Waters). The detailed sample preparation protocol and complete UPLC coupled with tandem mass spectrometry parameters are provided in Multimedia Appendix 1. Raw data processing, including peak integration and metabolite quantification, was conducted using the QuanMET software (version 2.0; Metabo-Profile). Quality control (QC) samples were analyzed intermittently to ensure analytical consistency.

### Standardization Measures: Ensuring Data Consistency Through Training and Reliability Checks

To avoid multicenter data bias, the following standardization measures were implemented before and during the study.

#### Unified Training

Specific training sessions were conducted, covering (1) interpretation of the methods and inclusion and exclusion criteria (eg, American Society of Anesthesiologists grade determination standards and MMSE scoring details), (2) demonstration of scale administration procedures (eg, step-by-step demonstration of the Chinese MMSE and CAM), and (3) case exercises (all involved health care professionals [including research nurses and psychiatrists] practiced independent scoring, followed by group review of discrepancies to unify judgment standards).

#### Platform Standardization

Metabolomics analysis was performed by Metabo-Profile. All samples were preprocessed according to a unified workflow (High-Throughput Targeted Metabolomics Platform section). One QC sample was inserted every 12 samples to ensure interbatch consistency of the assay results (coefficient of variation<30%).

### Blinding Design: Assessors Were Double Blinded to Metabolomics Results to Avoid Diagnostic Bias

To prevent knowledge of metabolomics results from influencing cognitive assessments or POD diagnosis, this study used an assessor-analyst double-blinding design.

#### Assessor Blinding

The psychiatrists and research nurses responsible for MMSE and CAM assessments were only aware of the patients’ clinical information (eg, age and surgery type) and were completely unaware of the patients’ preoperative serum metabolomics results (eg, differential metabolite levels and whether they belonged to a potential high-risk POD group). Metabolomics testing was performed by an independent laboratory. The test results were provided only to the statistical analyst after all cognitive assessments were completed and POD diagnoses were finalized. Only then were the metabolomics data and clinical outcomes merged for model building and analysis.

#### Statistical Analyst Blinding

Before modeling, the statistical analyst received only anonymized metabolomics data (patient IDs replaced with random numbers) and anonymized clinical outcomes (1=POD; 0=non-POD). The analyst was unaware of the patients’ specific clinical information to avoid subjective selection of feature variables.

### Machine Learning Model Development

#### Hyperparameter Tuning

Model hyperparameters were optimized via 7-fold cross-validation with grid search. Least absolute shrinkage and selection operator (LASSO) regression (λ) was tuned using 10-fold cross-validation (log(λ): −10 to 2). Random forest (RF) used 500 trees with mtry tuned among {2,9,16}. Gradient boosting (Extreme Gradient Boosting) was optimized for tree number (50-150), depth (1-5), learning rate (0.01-0.1), and minimum node size (minimum number of observations in the trees’ terminal nodes: 1-5). Logistic regression (LR) required no tuning.

#### Class Imbalance Handling

##### Overview

The POD-to–non-POD ratio was approximately 1:3.6. We used the area under the receiver operating characteristic (ROC) curve (AUC) as the primary metric due to its robustness to class imbalance. The test set achieved an AUC for the precision-recall (PR) curve of 0.966, confirming good minority-class identification.

##### Model Calibration

Calibration plots showed good agreement between predicted probabilities and observed outcomes for the final LR model.

##### Software

Analysis used R (version 4.3.2; R Foundation for Statistical Computing) with *caret* (version 6.0-94) for model training and tuning and *ggplot2* (version 3.5.0) for visualization.

##### Comprehensive Model Evaluation

The model’s performance was comprehensively evaluated beyond discrimination. The overall model performance was quantified using the Brier score. Calibration was assessed visually using calibration plots and numerically by calculating the calibration in the large (intercept) and calibration slope. The clinical utility of the model was evaluated using decision curve analysis, which quantifies the net benefit across a range of probability thresholds. Given the class imbalance (POD prevalence of approximately 21.5%), PR curves and the average precision (AP) score were also analyzed alongside ROC curves to provide a more informative assessment of the model’s performance in identifying the minority class (POD).

### Statistical Analysis

#### Overview

Demographic and surgical data were analyzed using SPSS (version 26.0; IBM Corp). For continuous variables, normality was assessed via the Kolmogorov-Smirnov test; normally distributed data were analyzed via one-way ANOVA, and nonnormally distributed data were analyzed via the Kruskal-Wallis *H* test. For categorical variables, group differences were evaluated using the chi-square test (significance threshold: *P*<.05).

Metabolomics data underwent multivariate and univariate analyses on the iMAP platform (version 1.0; Metabo-Profile). For multivariate analysis, principal component analysis for dimensionality reduction and outlier detection was conducted, with partial least squares discriminant analysis and orthogonal partial least squares discriminant analysis to maximize intergroup discrimination (threshold: Variable Importance in Projection>1.0). For univariate analysis, differential metabolites were screened via 2-tailed *t* test and Mann-Whitney *U* test (threshold: *P*<.05).

RF and LASSO regression identified potential biomarkers [28]. The predictive performance of biomarkers was evaluated using gradient boosting, LR, and RF models, with ROC and PR curves generated to assess diagnostic accuracy.

A nested cross-validation approach was used to mitigate overfitting and robustly assess model generalizability. The cohort (N=260) was initially randomly split into a training set (70%; n=182) and a hold-out test set (30%; n=78). Model development and hyperparameter tuning were confined strictly to the training set using 7-fold cross-validation. In this inner loop, the training set was partitioned into 7 folds; the model was iteratively trained on 6 folds and validated on the remaining fold. The model with the best average performance across the 7 folds (LR) was retrained on the entire training set, and its final performance was evaluated on the independent test set. This strategy prevents information leakage and ensures an unbiased performance estimate.

The final analytical cohort comprised 260 patients, including 56 (21.5%) with POD. For the development of the machine learning model using the 16 selected metabolites, the event per variable (EPV) ratio was 56/16 = 3.5. While an EPV of 10 is often recommended for ideal stability in predictive modeling, an EPV greater than 3 is generally considered acceptable in exploratory biomarker discovery studies, particularly in fields such as metabolomics where prior data are limited [29]. Furthermore, the obtained AUC of 0.855 with a 95% CI of 0.800 to 0.910 indicates a reasonably precise estimate of the model’s discriminative ability. This precision, evidenced by the CI width, supports the adequacy of the sample size for the primary objective of model development and initial internal validation within this cohort.

The 95% CIs for the AUC were calculated using the DeLong method [30], which accounts for the pairwise correlation between sensitivity and specificity across all possible thresholds.

#### Data Preprocessing and QC

Metabolites with more than 20% of missing values were excluded. In this study, all 201 targeted metabolites exhibited missing rates below 2%, and thus, no metabolites required exclusion. Given the minimal missingness, no imputation was performed to preserve data integrity. Batch effects were corrected using QC-based robust locally estimated scatterplot smoothing signal correction implemented in the QuanMET software. For class imbalance between the POD and non-POD groups (POD-to–non-POD ratio of approximately 1:3.6), class weight adjustment was applied to all machine learning models, with weights set inversely proportional to class frequencies.

## Results

### Summary Workflow Diagram to Illustrate the Complete Research Logic

A schematic overview summarizing the complete study workflow is provided in Figure S1 in Multimedia Appendix 1. This diagram visually integrates the key stages of the research, from participant recruitment and data collection to metabolomics profiling, machine learning model development, and the final predictive outcome for POD.

### Comparison of General Characteristics Between the POD and Non-POD Groups

**Figure 1.**
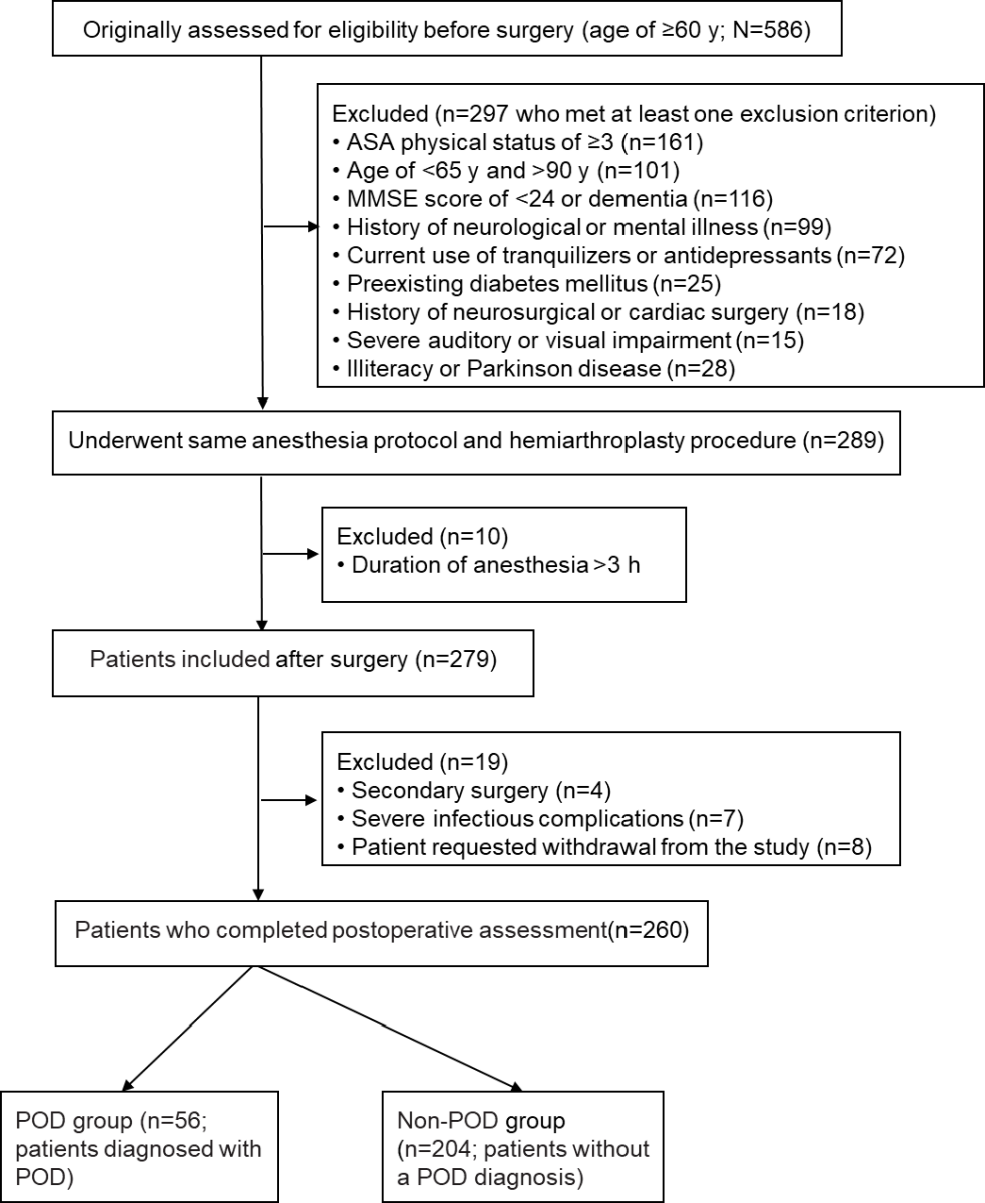
Flowchart of patient screening (final analytical cohort: N=260 older adult patients with hip fracture undergoing hemiarthroplasty; postoperative delirium [POD] group: n=56; non-POD group: n=204). ASA: American Society of Anesthesiologists; MMSE: Mini-Mental State Examination.

The study flowchart (Figure 1) illustrates the patient enrollment process in this multicenter observational cohort study. Ultimately, 260 patients met the inclusion criteria and completed the study protocol. Of these 260 patients, 56 (21.5%) were diagnosed with POD, constituting the POD group, whereas 204 (78.5%) without a POD diagnosis were categorized as the non-POD group. As shown in Table 1, no significant differences were observed between the 2 groups in demographic characteristics, surgical duration, or anesthesia time.

**Table 1.** Demographic characteristics of the postoperative delirium (POD) and non-POD groups.

Characteristic	POD group (n=56)	Non-POD group (n=204)	*P* value
Age (y), mean (SD)	73.8 (6.8)	72.9 (7.1)	.39
Sex, n (%)			.86
Female	35 (62.5)	128 (62.7)	
Male	21 (37.5)	76 (37.3)	
BMI (kg/m^2^), mean (SD)	20.33 (3.7)	20.87 (3.9)	.22
Education (y), mean (SD)	7.6 (4.1)	7.2 (3.9)	.40
ASA^a^ grade, n (%)			.88
1	25 (44.6)	89 (43.6)	
2	31 (55.4)	115 (56.4)	
Preoperative MMSE^b^ score (0-30), mean (SD)	27.49 (1.9)	27.83 (2.0)	.24
Preoperative CCI^c^, mean (SD)	1.9 (0.7)	1.8 (0.9)	.21
Operation duration (min), mean (SD)	93 (38)	88 (39)	.30
Anesthesia duration (min), mean (SD)	119 (35)	117 (39)	.68

a ASA: American Society of Anesthesiologists.

b MMSE: Mini-Mental State Examination.

c CCI: Charlson Comorbidity Index.

### Ethical Reporting of Patient Flow and Handling of Attrition

The patient flow throughout the study is detailed in Figure 1. All cases of attrition were meticulously documented in the study case report forms, including the specific reason and time point of withdrawal. This documentation was reviewed and signed by the principal investigator at each site to ensure adherence to ethical standards.

Data from patients who dropped out were handled as follows:

- Patients excluded for medical reasons (n=11): data collected from these patients prior to their exclusion (eg, preoperative blood samples and baseline demographics) were not included in the final statistical analysis or model development. This conservative approach was taken to prevent the introduction of confounding factors related to their severe postoperative medical complications, which are distinct from the pathophysiology under investigation and could distort the association between metabolic profiles and POD.
- Patients lost to follow-up (n=8): as these patients withdrew consent for continued participation in the study protocol postoperatively, no further data were collected. Any preliminary data obtained prior to withdrawal were not used in the analysis, respecting patient autonomy and the ethical principle of voluntary participation.

This rigorous approach to reporting patient flow and handling missing data ensures the transparency, reproducibility, and ethical integrity of our research findings. The final analytical cohort (N=260) represents a complete-case dataset for the planned metabolomics and machine learning analyses.

### Differences in Preoperative Serum Metabolites Identified Using a High-Throughput Targeted Metabolomics Platform Between the POD and Non-POD Groups

**Figure 2.**
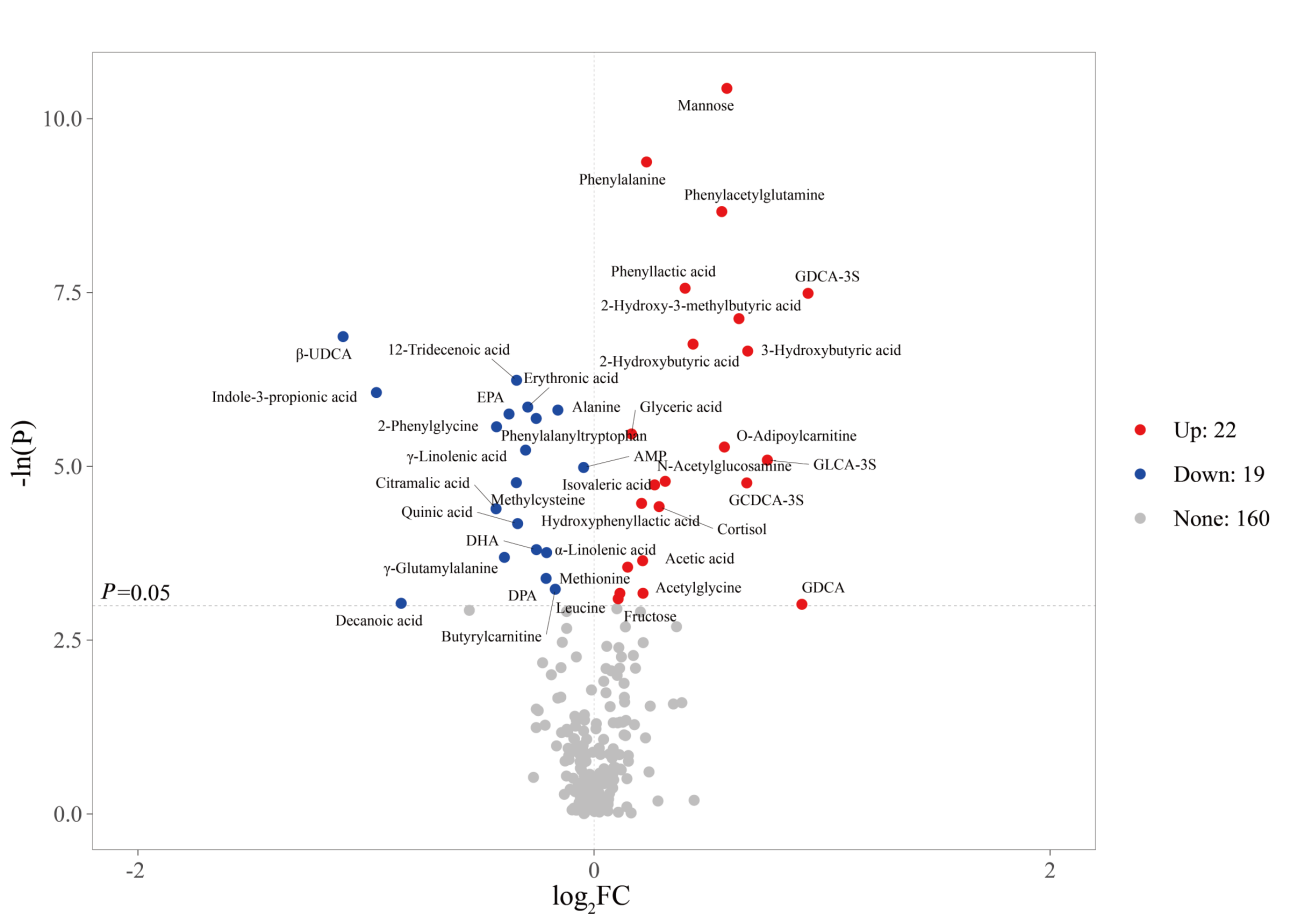
Volcano plot of differentially abundant preoperative serum metabolites between the postoperative delirium (POD) and non-POD groups (41 significant metabolites identified). β-UDCA: β-ursodeoxycholic acid; AMP: adenosine monophosphate; DHA: docosahexaenoic acid; DPA: docosapentaenoic acid; EPA: eicosapentaenoic acid; GCDCA-3S: glycochenodeoxycholic acid 3-sulfate; GDCA: glycodeoxycholic acid; GDCA-3S: glycodeoxycholic acid 3-sulfate; GLCA-3S: glycolithocholic acid 3-sulfate; log2FC: log 2 fold change.

To compare metabolite profiles, we used UPLC coupled with triple quadrupole mass spectrometry for absolute quantification of 201 metabolites. As shown in Figure 2, absolute quantification of 201 metabolites revealed 41 (20.4%) significantly differentially expressed metabolites between the POD and non-POD groups (*P*<.05). These differential metabolites were primarily enriched within 4 core metabolic domains (amino acid metabolism, energy metabolism, lipid metabolism, and bile acid metabolism), suggesting that POD development is closely associated with disruptions in these key biological pathways.

**Figure 3.**
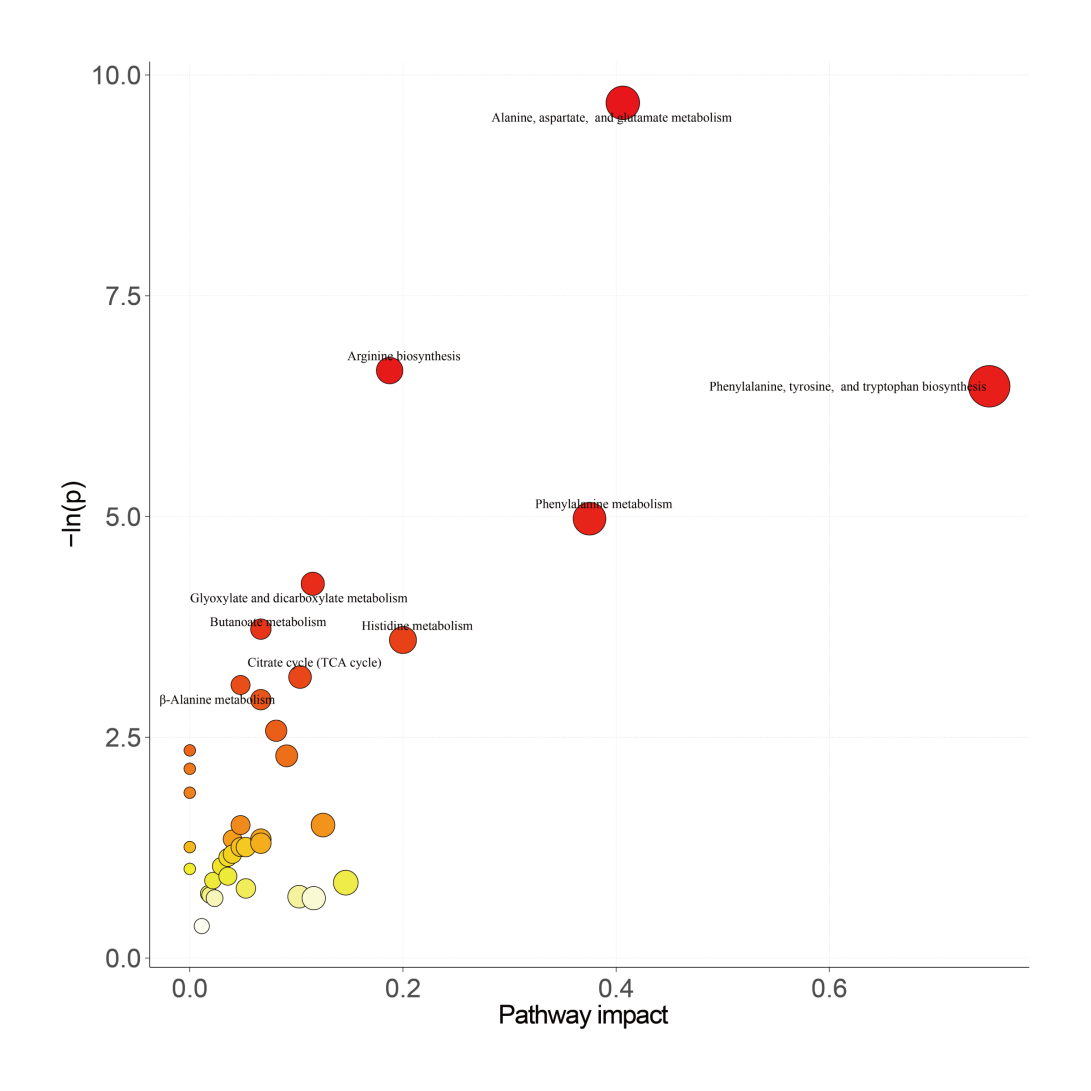
Bubble plot of Kyoto Encyclopedia of Genes and Genomes pathway enrichment analysis for the differential metabolites (core enrichment in 4 metabolic pathways; *P*<.05). TCA: tricarboxylic acid.

Kyoto Encyclopedia of Genes and Genomes pathway enrichment analysis further confirmed that these differential metabolites were significantly enriched in key pathophysiological pathways, including alanine, aspartate, and glutamate metabolism (neurotransmitter synthesis); phenylalanine, tyrosine, and tryptophan biosynthesis (precursors for dopamine and serotonin); and the citrate cycle (TCA cycle and core energy metabolism; Figure 3).

### Machine Learning–Powered Discovery of Specific Metabolic Biomarkers for Predictive Modeling of POD

**Figure 4.**
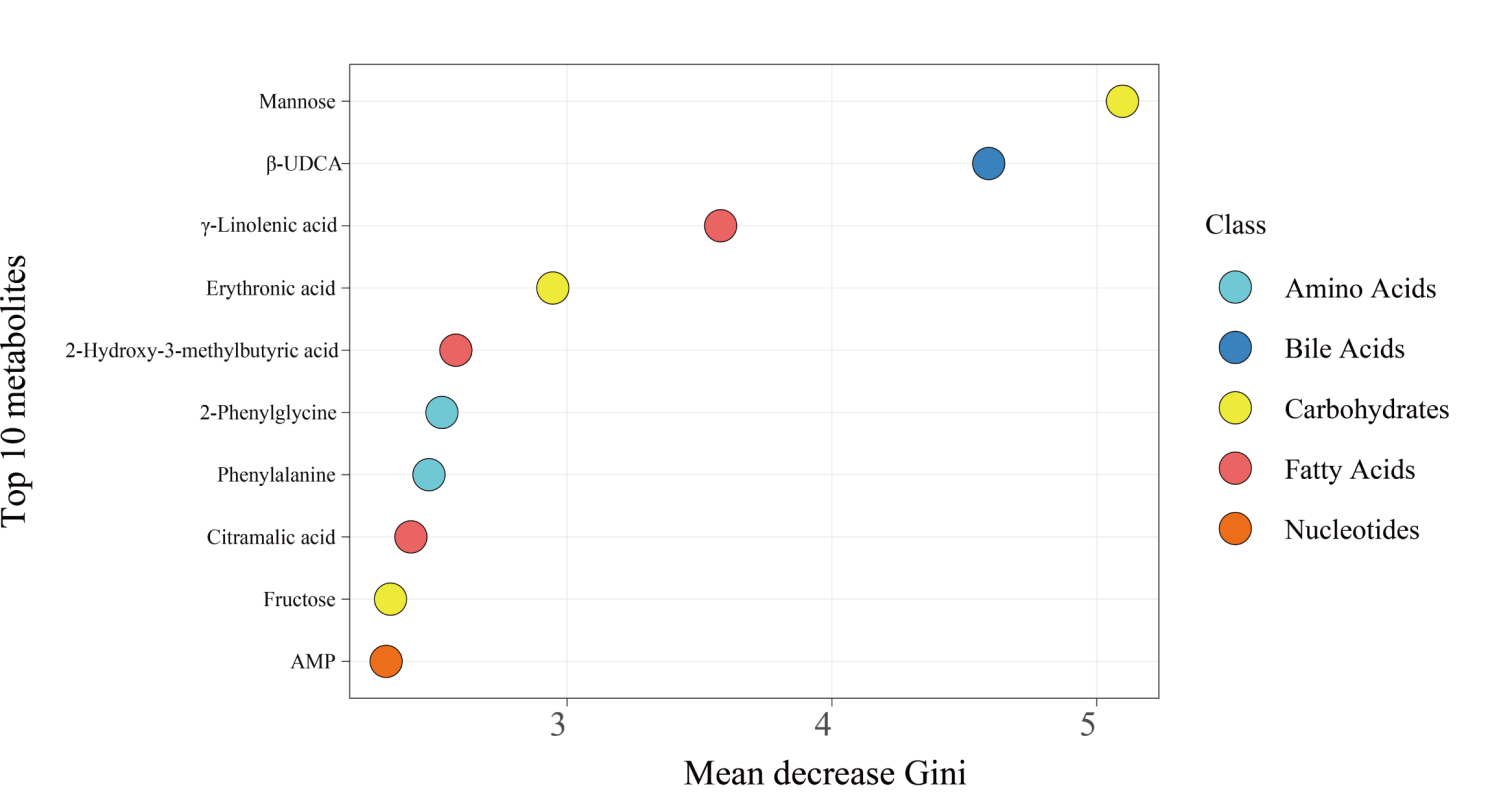
Top 10 core predictive metabolites screened using the random forest algorithm (ranked by Gini importance). β-UDCA: β-ursodeoxycholic acid; AMP: adenosine monophosphate.

**Figure 5.**
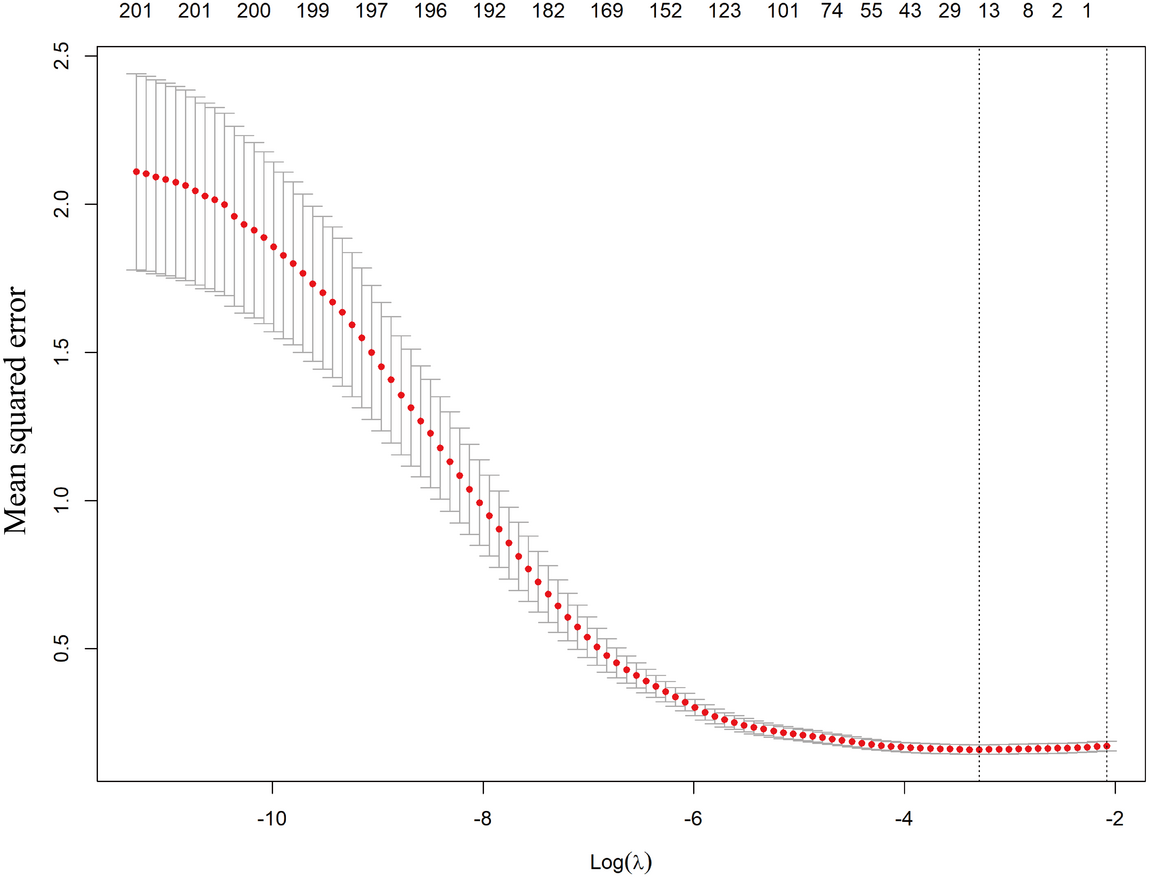
Feature selection plot for differential metabolites using least absolute shrinkage and selection operator regression (12 key metabolites selected at optimal λ).

**Figure 6.**
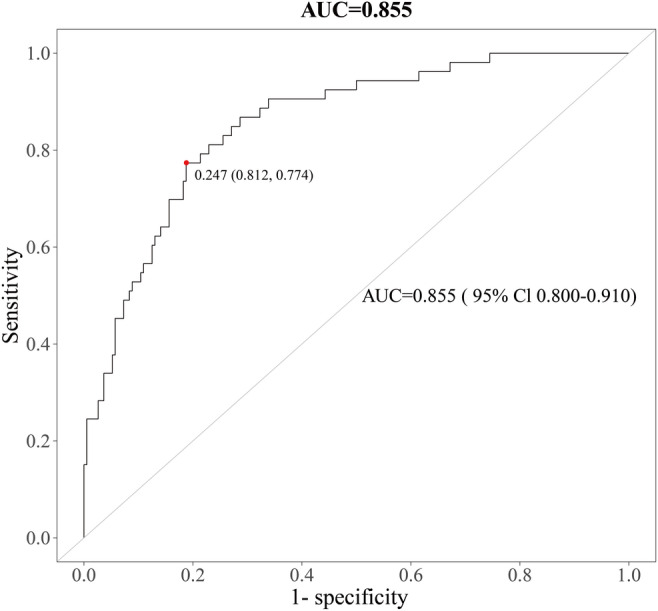
Receiver operating characteristic (ROC) curve of the logistic regression model for predicting postoperative delirium based on 16 core metabolites in the overall cohort (area under the ROC curve [AUC]=0.855, 95% CI 0.800‐0.910).

By performing a union operation on the top 10 differentially abundant metabolites identified through RF model screening (Figure 4) and LASSO regression–based feature selection (Figure 5), we obtained 16 distinct metabolites (see Table S1 in Multimedia Appendix 1 for the complete list). These biomarkers span the aforementioned 4 core metabolic domains and collectively form the feature set for predictive modeling. Subsequently, predictive models were constructed using these 16 metabolites via gradient boosting, LR, and RF algorithms. Among these, the LR model demonstrated strong predictive performance, achieving an AUC of 0.855 (95% CI 0.800‐0.910; Figure 6).

### Randomized Cohort Partition Into Training and Test Sets for Machine Learning Validation of the POD Prediction Model

**Figure 7.**
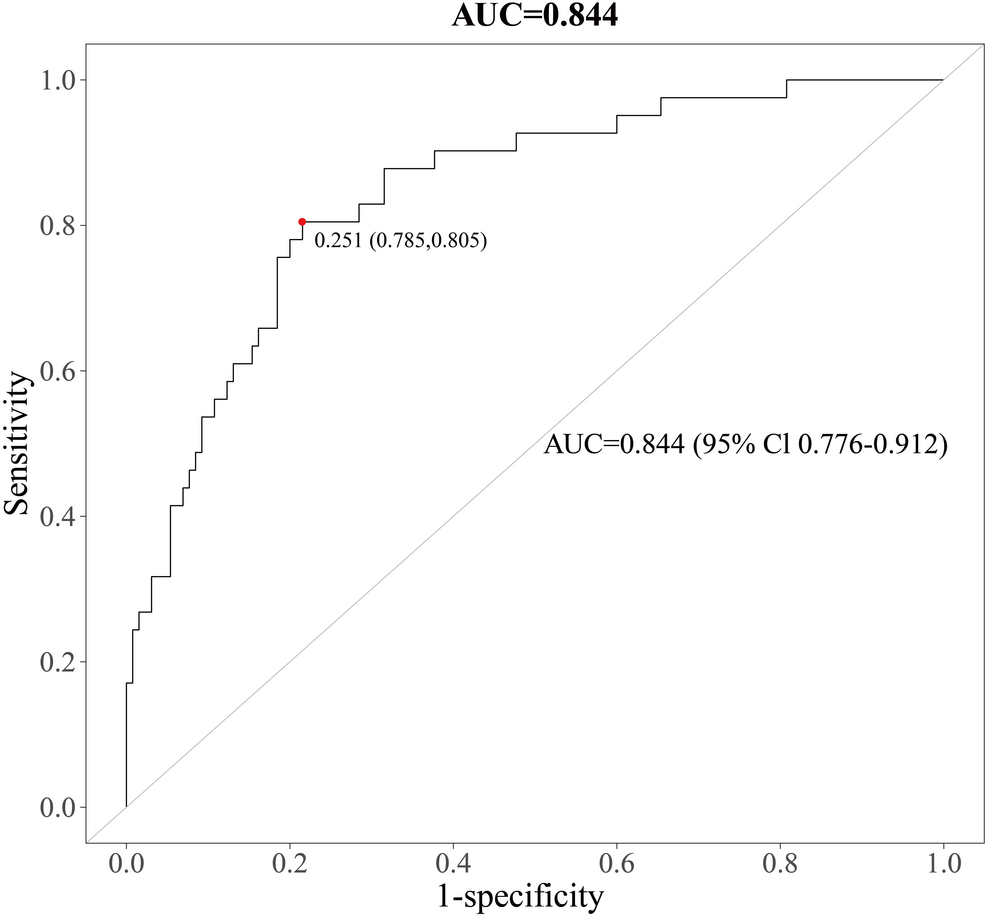
Receiver operating characteristic (ROC) curve of the logistic regression model based on 16 core metabolites in the training cohort (area under the ROC curve [AUC]=0.844, 95% CI 0.776‐0.912).

**Figure 8.**
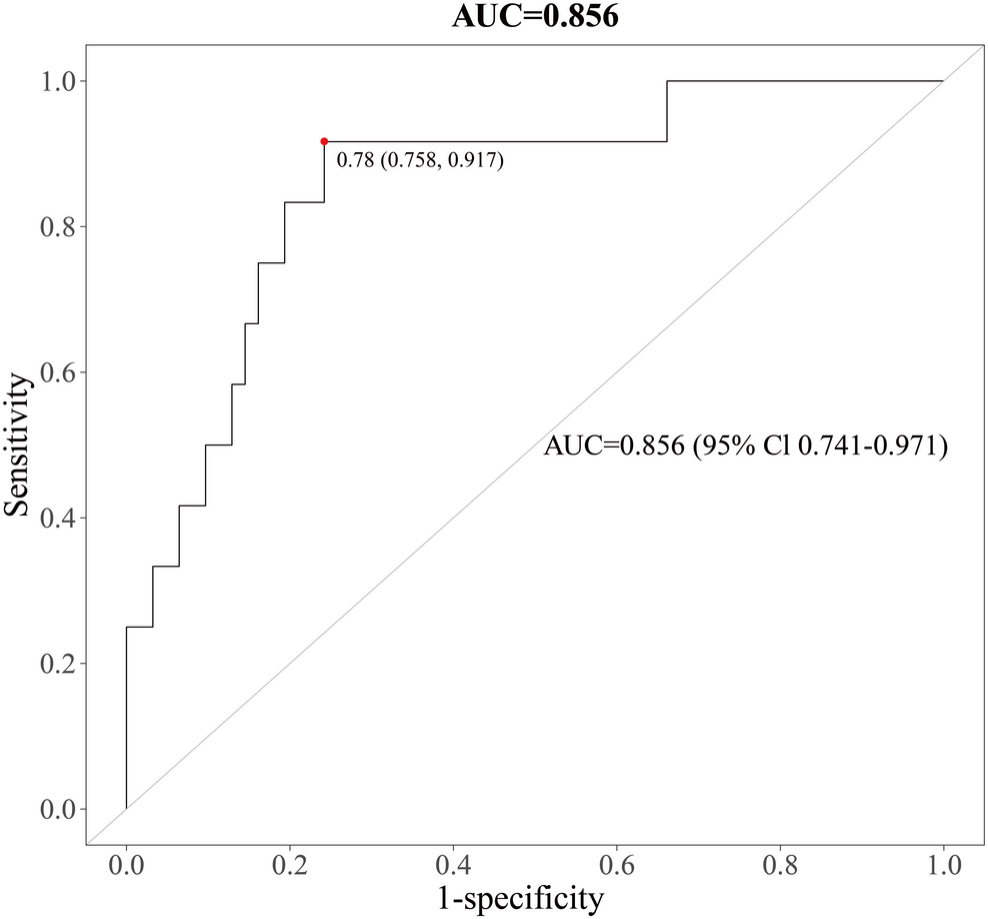
Receiver operating characteristic (ROC) curve of the logistic regression model based on 16 core metabolites in the independent test cohort (area under the ROC curve [AUC]=0.856, 95% CI 0.741–0.971).

Total samples were randomly partitioned into training and testing sets (7:3 ratio) via computerized randomization algorithms. Differential metabolites underwent rigorous LR modeling, demonstrating robust predictive performance with AUC values of 0.844 (95% CI 0.776–0.912) in the training cohort (Figure 7) and 0.856 (95% CI 0.741–0.971) in the independent test cohort (Figure 8), meeting TRIPOD (Transparent Reporting of a Multivariable Prediction Model for Individual Prognosis or Diagnosis) guidelines for clinical prediction models.

### Comprehensive Evaluation of Model Performance: Discrimination, Calibration, and Clinical Utility

**Figure 9.**
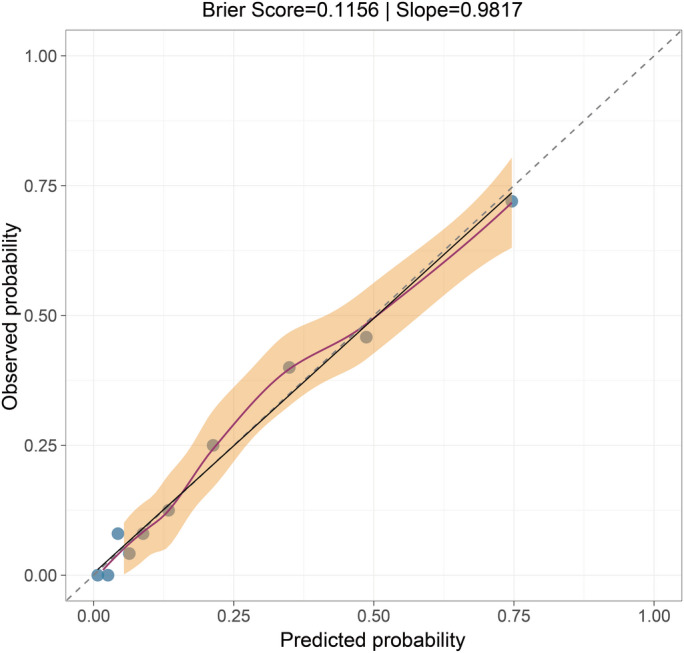
Calibration performance of the postoperative delirium prediction model, showing excellent fit (as indicated by the Brier score and calibration slope) between predictions and observations.

The final LR model was subjected to a comprehensive evaluation on the independent test set (n=78). The model demonstrated good overall accuracy, with a Brier score of 0.1156. The calibration plot (Figure 9) indicated excellent agreement between predicted probabilities and observed outcomes, with a calibration slope of 0.9817.

**Figure 10.**
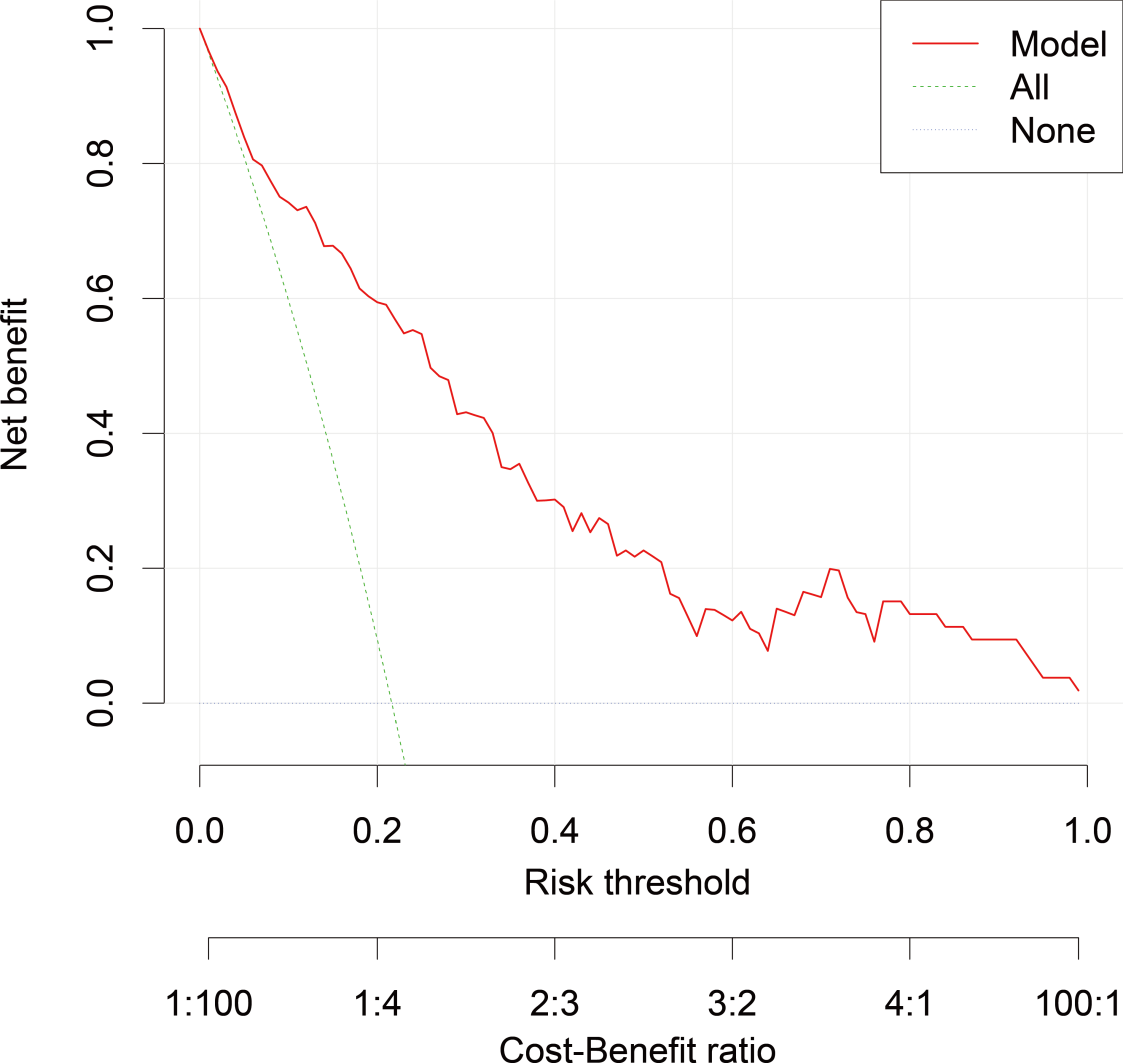
Decision curve analysis demonstrating the clinical utility of the metabolomics model, which provides a positive net benefit over a wide range of probability thresholds for postoperative delirium prediction.

Decision curve analysis (Figure 10) revealed that using the metabolomics model for clinical decision-making provided a superior net benefit compared to strategies of intervening on all patients or intervening on no patients across a wide range of clinically reasonable probability thresholds (approximately 10% to 60%). This underscores the potential clinical utility of the model for targeting preventive interventions.

**Figure 11.**
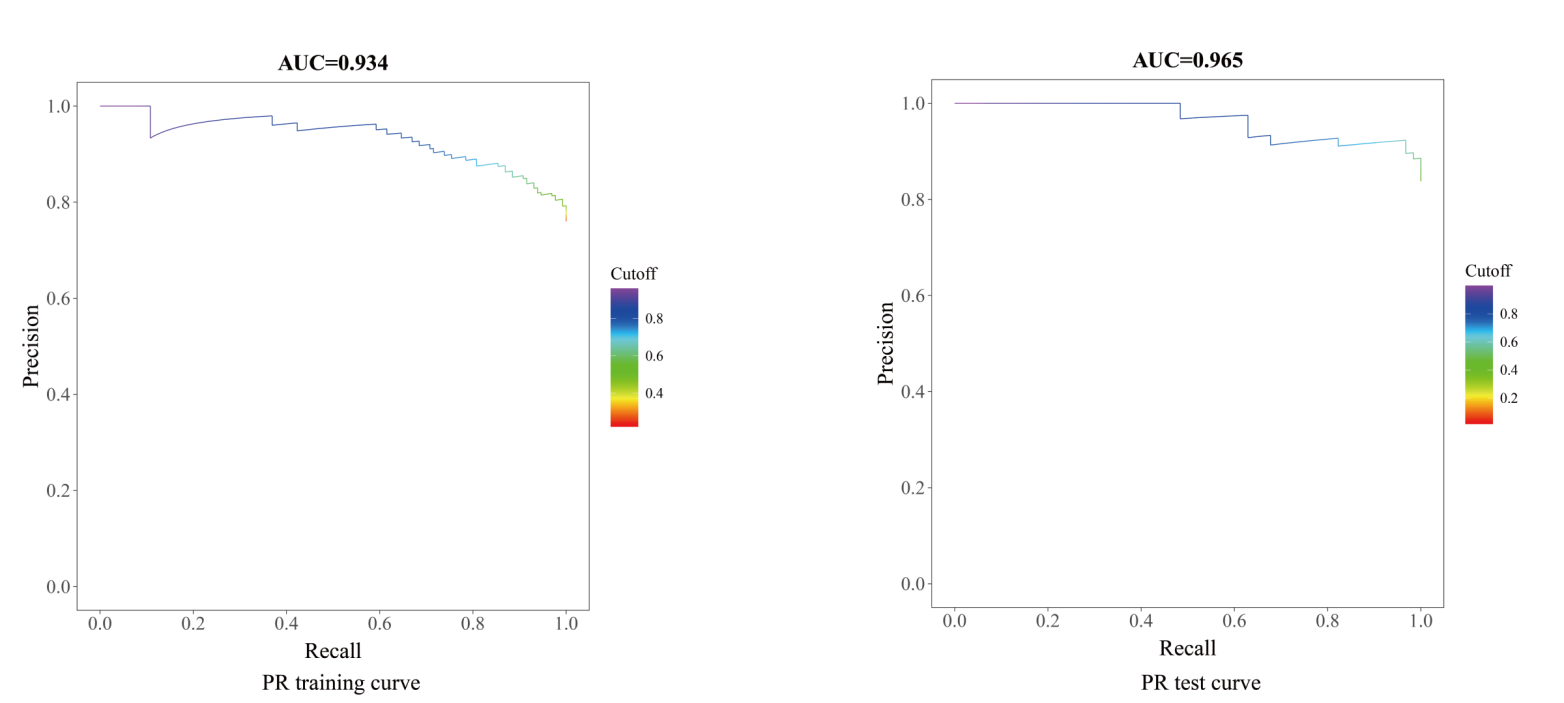
Precision-recall (PR) curves for the postoperative delirium prediction model. The high average precision scores on both the training and test sets confirm robust performance in identifying the minority class despite data imbalance. AUC: area under the receiver operating characteristic curve.

In light of the class imbalance, PR curves were analyzed (Figure 11). The model achieved an AP of 0.965 on the test set (training set AP=0.934). This exceptionally high AP, in the context of a 21.5% (56/260) event rate, confirms the model’s robust capability to identify patients with POD with high precision and recall. The PR curve is particularly informative for this imbalanced classification task as it focuses on the performance regarding the positive class (POD), unlike the ROC curve, which can be overly optimistic under class imbalance.

**Figure 12.**
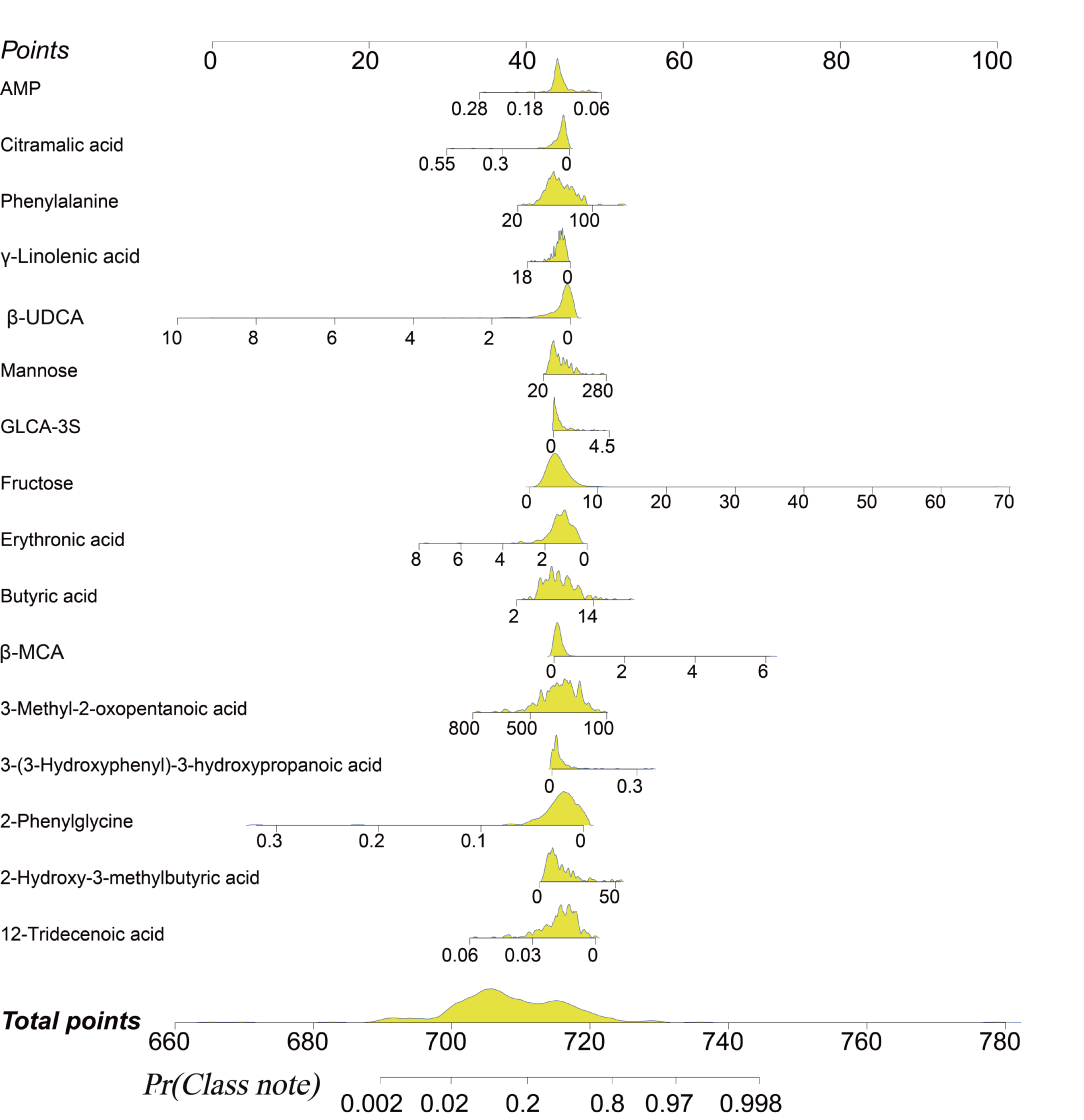
Nomogram for individualized postoperative delirium (POD) risk prediction derived from the logistic regression model incorporating 16 preoperative metabolites. The total points from all metabolites correspond to an individual’s predicted POD probability. β-MCA: β-muricholic acid; β-UDCA: β-ursodeoxycholic acid; AMP: adenosine monophosphate; GLCA-3S: glycolithocholic acid 3-sulfate.

To facilitate clinical application, a nomogram was constructed based on the final LR model (Figure 12), allowing for the visual calculation of an individual patient’s POD risk using the 16 preoperative metabolite levels.

## Discussion

### Principal Findings

POD, a severe neurological complication in older adult patients, is associated with neuroinflammation, oxidative stress, energy metabolism dysfunction, and cholinergic system imbalance [31]. Using a multicenter high-throughput targeted metabolomics approach combined with machine learning, we identified 16 differentially expressed metabolites as key biomarkers for constructing a POD prediction model.

#### Energy and Carbohydrate Metabolism

Altered levels of fructose and mannose indicate glucose metabolism dysregulation. Older adult patients often have insulin resistance, and postoperative stress exacerbates the imbalance between glycolysis and oxidative phosphorylation, leading to insufficient energy supply in the CNS [32]. Adenosine monophosphate, a key energy sensor, reflects mitochondrial dysfunction—overactivation of the adenosine monophosphate–activated protein kinase pathway under energy stress induces neuronal energy crisis linked to cognitive impairment in delirium [33].

#### Amino Acid Metabolism Dysregulation

Accumulation of phenylalanine, possibly due to reduced hepatic metabolism in older adults, disrupts the synthesis of catecholamine neurotransmitters (eg, dopamine and norepinephrine), exacerbating neuropsychiatric symptoms in POD [34]. 2-Phenylglycine, a phenylalanine derivative, suggests dysregulation of aromatic amino acid metabolism associated with oxidative stress–induced neurotoxicity [35]. 3-Methyl-2-oxopentanoic acid, an intermediate of branched-chain amino acid metabolism, indicates dysfunction of the mitochondrial branched-chain ketoacid dehydrogenase complex, leading to CNS energy deficiency and neuroinflammatory cascades [35].

#### Lipid and Bile Acid Metabolism Disorders

Abnormal γ-linolenic acid levels, a polyunsaturated fatty acid, reflect membrane lipid homeostasis disruption, promoting proinflammatory mediator synthesis and neuroinflammation—a core mechanism of POD [36]. Butyric acid, a gut microbiota–derived short-chain fatty acid, regulates microglia anti-inflammatory function via the gut-brain axis; postoperative gut dysbiosis in older adult patients may reduce butyric acid, weakening its suppression of neuroinflammation [37]. β-Ursodeoxycholic acid and β-muricholic acid are associated with altered blood-brain barrier permeability; bile acids regulate neuroinflammation through the farnesoid X receptor and Takeda G protein–coupled receptor 5, and their imbalance activates astrocytes and impairs synaptic function [38]. Elevated glycolithocholic acid 3-sulfate suggests reduced hepatic detoxification, leading to toxic bile acid accumulation in the brain and neuronal injury [39].

#### Organic Acids and Oxidative Stress

Increased erythronic acid is linked to polyol pathway activation under oxidative stress, causing neuronal osmotic imbalance and reduced antioxidant capacity [40]. Citramalic acid, a TCA cycle intermediate, indicates mitochondrial citrate transporter dysfunction, leading to energy metabolism disorders and excessive reactive oxygen species production, intensifying neuronal oxidative damage [41]. 2-Hydroxy-3-methylbutyric acid, a leucine metabolite, is associated with enhanced catabolism under postoperative stress, indirectly reflecting the impact of systemic inflammatory load on the CNS [42]. Accumulation of 3-(3-hydroxyphenyl)-3-hydroxypropanoic acid, a phenylalanine metabolite, may result from abnormal phenylalanine hydroxylase activity, increasing neurotoxic metabolite production [43]. The role of 12-tridecenoic acid, a long-chain fatty acid, remains unclear but may involve membrane lipid regulation or inflammatory mediator synthesis, warranting further investigation [44].

#### Integration of Innovation Frameworks and Translational Implications

This study presents a distinctive research paradigm that integrates core principles from modern innovation frameworks. Our approach aligns with design thinking by centering on the clinical challenge of POD and iteratively developing an artificial intelligence–metabolomics model, with the resulting 16-metabolite panel representing a validated minimum viable product. This study follows the Lean Startup ethos through stringent RF- and LASSO-based feature selection to build a parsimonious yet powerful predictor (AUC>0.85), avoiding overengineering while enhancing clinical feasibility. Ultimately, this tool advances precision medicine by enabling individualized risk quantification based on objective metabolic profiles, facilitating a shift from reactive treatment to proactive prevention and demonstrating high translational potential in perioperative care.

This study also has limitations. Despite rigorous internal validation, the model requires validation in an external, geographically distinct cohort as our multicenter sample was drawn solely from Shanghai, potentially limiting generalizability. The model’s scalability, while aided by its objective metabolic inputs, faces challenges including platform accessibility, ethnic variations in metabolic baselines requiring recalibration, and integration into diverse clinical workflows. Future work will focus on external validation across broader populations and surgical subtypes to advance clinical translation. Moreover, we agree that the clinical translation of our metabolomics-only model faces practical constraints regarding cost, turnaround time, and standardization. More fundamentally, as POD is multifactorial, a model relying solely on metabolites cannot incorporate key clinical risk factors such as frailty or comorbidity burden. To address these limitations, our primary future plan is to integrate this metabolomics panel with essential clinical variables to build a multimodal model. This integration is expected to enhance clinical practicality and robustness while maintaining high predictive performance. Furthermore, we have planned a prospective, external validation study to rigorously assess the model’s generalizability.

### Conclusions

This multicenter metabolomics study identified 16 differentially expressed metabolites involved in core pathological pathways of POD in older adult patients, including energy metabolism, amino acid homeostasis, bile acid regulation, and oxidative stress. These metabolites provide multidimensional biomarkers for early POD prediction and novel targets for interventions (eg, improving mitochondrial function and regulating gut microbiota). The machine learning–based prediction model has potential for integration into perioperative management to achieve precise POD prevention through dynamic metabolic profiling. However, to facilitate its clinical translation, future studies should focus on (1) integrating these metabolites with readily available key clinical variables to develop a more universally applicable and practical multimodal prediction tool; (2) rigorously validating the refined model in independent, multiregional external cohorts to assess its real-world performance; and (3) further elucidating the potential causal associations between these metabolites and the development of POD, and exploring their specific roles across different surgical subtypes.

## Supplementary material

10.2196/78495 Multimedia Appendix 1 Supplementary materials including detailed ultrahigh-performance liquid chromatography-mass spectrometry/mass spectrometry parameters, study workflow diagram, and complete list of 16 candidate metabolic biomarkers.

## Abbreviations

AP: average precision
AUC: area under the receiver operating characteristic curve
CAM: Confusion Assessment Method
CNS: central nervous system
EPV: event per variable
LASSO: least absolute shrinkage and selection operator
LR: logistic regression
MMSE: Mini-Mental State Examination
POD: postoperative delirium
PR: precision-recall
QC: quality control
RF: random forest
ROC: receiver operating characteristic
TCA: tricarboxylic acid
TRIPOD: Transparent Reporting of a Multivariable Prediction Model for Individual Prognosis or Diagnosis
UPLC: ultrahigh-performance liquid chromatography
