# Supplementary material for: Integrated High-Throughput Targeted Metabolomics and Machine Learning for Early Prediction and Prevention of Postoperative Delirium in Older Adult Surgical Patients: Prospective Multicenter Cohort Study
Source: JMIR Aging. 2026 Apr 30;9:e78495. doi: 10.2196/78495 (PMC13131831; doi:10.2196/78495)
Supplement: Multimedia Appendix 1 [file aging-v9-e78495-s001.pdf]

APPENDIX

SUPPLEMENTARY Method S1: Detailed Protocol for High-Throughput Targeted Metabolomics

1. Sample Preparation Protocol

Serum samples were processed using the Q300 Metabolite Assay Kit (Metabo-Profile Corp., Shanghai, China) according to the manufacturer's instructions with the following detailed steps:

a) **Aliquoting: 20 μL of each serum sample was transferred into a 96-well plate.**
b) **Protein Precipitation: 120 μL of ice-cold methanol containing internal standards was added to each well using an Eppendorf epMotion workstation (Eppendorf Inc., Hamburg, Germany).**
c) **Vortexing: The plate was sealed and vortexed vigorously for 5 minutes.**
d) **Centrifugation: The plate was centrifuged at 4°C and 4000 ×g for 30 minutes.**
e) **Derivatization: 20 μL of freshly prepared derivatization reagent was added to each well. The plate was resealed and incubated at 30°C for 60 minutes.**
f) **Dilution: 330 μL of ice-cold 50% methanol was added to each well.**
g) **Second Centrifugation: The plate was centrifuged again (4°C, 4000 ×g, 30 minutes).**
h) **Supernatant Transfer: 135 μL of supernatant was transferred to a new 96-well plate.**
i) **Internal Standard Addition: 10 μL of internal standard was added to each well of the new plate.**
j) **Calibration Standards: Derivatized standard solutions at gradient concentrations were loaded into the edge wells for calibration.**
k) **Analysis: The final plate was sealed for UPLC-MS/MS analysis.**

2. UPLC-MS/MS Instrument Parameters

- **Chromatographic System: Waters ACQUITY UPLC system coupled with a Xevo TQ-S mass spectrometer (Waters, Milford, MA, USA).**
- **Column: ACQUITY UPLC BEH C18 (1.7 μm, 2.1 mm × 100 mm; Waters).**
- **Column Temperature: 40°C**
- **Injection Volume: 5 μL**
- **Flow Rate: 0.4 mL/min**
- **Mobile Phase:**
    - **A: 0.1% formic acid in LC-MS grade water.**
    - **B: Acetonitrile:Isopropanol (7:3, v/v).**
- **Gradient Program:**

Time (min)	%B
0.0 - 1.0	5
1.0 - 5.0	5→30
5.0 - 9.0	30→50
9.0 - 11.0	50→78
11.0 - 13.5	78→95
13.5 - 14.0	95→100
14.0 - 16.0	100
16.0 - 16.1	100→5
16.1 - 18.0	5

- **Mass Spectrometric Conditions:**
  - **Ionization Modes: Electrospray Ionization (ESI), positive and negative**
  - **Capillary Voltage: 1.5 kV (Positive), 2.0 kV (Negative)**
  - **Source Temperature: 150°C**
  - **Desolvation Temperature: 550°C**
  - **Data Acquisition: Multiple Reaction Monitoring (MRM)**

3. Quality Control and Data Processing

- **Sample Randomization: Samples were injected in random order.**
- **Quality Control (QC): A pooled QC sample was analyzed every 12 injections.**
- **Data Processing: Raw data were processed using QuanMET software (v2.0) for peak integration and quantification. Batch effects were corrected via QC-RLSC.**

SUPPLEMENTARY Figure S1. Comprehensive schematic of the study workflow for predicting postoperative delirium (POD).
The diagram outlines the five-stage research process:

Module 1 (Study Design & Cohort Finalization): Illustrates participant screening, application of inclusion/exclusion criteria, and final cohort establishment (n=260).

Module 2 (Data & Biospecimen Collection): Details the acquisition of clinical data and preoperative serum samples, followed by high-throughput targeted metabolomic profiling (quantifying 201 metabolites).

Module 3 (Data Preprocessing & Feature Selection): Shows the steps of data normalization, missing value imputation, and recursive feature elimination using Random Forest (RF) and LASSO regression, which identified 16 core predictive metabolites.

Module 4 (Model Construction & Validation): Depicts the construction of machine learning models, their internal validation via 7-fold cross-validation (training set AUC = 0.844), and subsequent evaluation on an independent test set (test set AUC = 0.856). This module also includes the biological interpretation of results through metabolic pathway enrichment analysis.

Module 5 (Study Conclusion): Summarizes the core finding: the development and performance of an integrative predictive model for POD risk, achieving an overall AUC of 0.855.

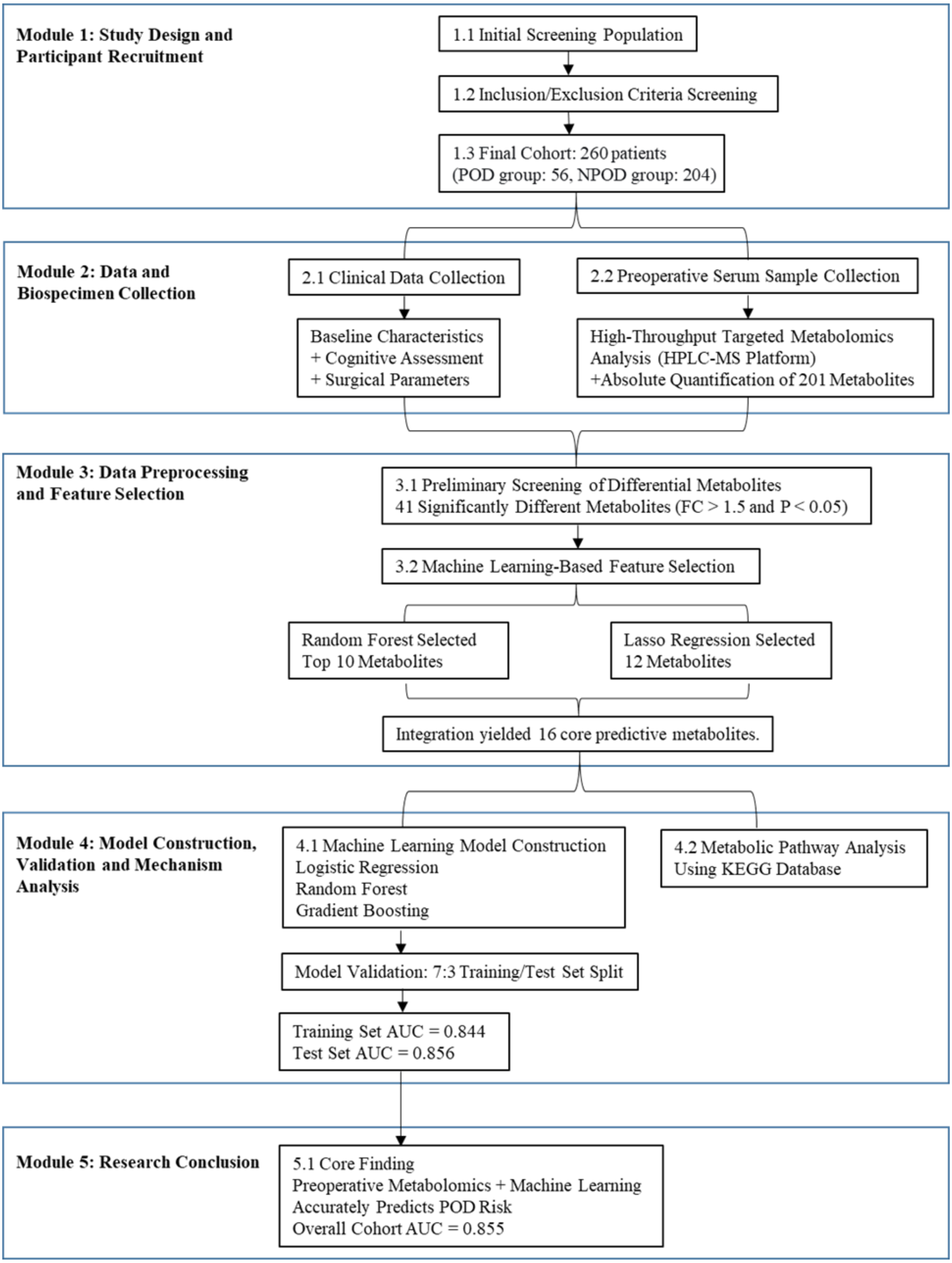
Module 1: Study Design and Participant Recruitment
1.1 Initial Screening Population
1.2 Inclusion/Exclusion Criteria Screening
1.3 Final Cohort: 260 patients (POD group: 56, NPOD group: 204)
Module 2: Data and Biospecimen Collection
2.1 Clinical Data Collection
2.2 Preoperative Serum Sample Collection
Baseline Characteristics + Cognitive Assessment + Surgical Parameters
High-Throughput Targeted Metabolomics Analysis (HPLC-MS Platform) +Absolute Quantification of 201 Metabolites
Module 3: Data Preprocessing and Feature Selection
3.1 Preliminary Screening of Differential Metabolites 41 Significantly Different Metabolites (FC > 1.5 and P < 0.05)
3.2 Machine Learning-Based Feature Selection
Random Forest Selected Top 10 Metabolites
Lasso Regression Selected 12 Metabolites
Integration yielded 16 core predictive metabolites.
Module 4: Model Construction, Validation and Mechanism Analysis
4.1 Machine Learning Model Construction Logistic Regression Random Forest Gradient Boosting
4.2 Metabolic Pathway Analysis Using KEGG Database
Model Validation: 7:3 Training/Test Set Split
Training Set AUC = 0.844 Test Set AUC = 0.856
Module 5: Research Conclusion
5.1 Core Finding Preoperative Metabolomics + Machine Learning Accurately Predicts POD Risk Overall Cohort AUC = 0.855

SUPPLEMENTARY Table S1. The 16 metabolites were selected through RF (Random Forest) - and LASSO (Least Absolute Shrinkage and Selection Operator) -based recursive feature elimination, grouped by Category.

Metabolite Category	Metabolite Name
**Fatty Acids**	12-Tridecenoic acid
	gamma-Linolenic acid
	Butyric acid
**Amino Acids & Derivatives**	Phenylalanine
	2-Phenylglycine
	3-Methyl-2-oxopentanoic acid
	3-(3-Hydroxyphenyl)-3-hydroxypropanoic acid
**Organic Acids**	2-Hydroxy-3-methylbutyric acid
	Erythronic acid
	Citramalic acid
**Carbohydrates**	Fructose
	Mannose
**Bile Acids**	β-Muricholic acid (bMCA)
	β-Ursodeoxycholic acid (bUDCA)
	Glycolithocholic acid-3-sulfate (GLCA-3S)
**Nucleotide**	Adenosine monophosphate (AMP)
